# A synergistic role of convalescent plasma and mesenchymal stem cells in the treatment of severely ill COVID-19 patients: a clinical case report

**DOI:** 10.1186/s13287-020-01802-8

**Published:** 2020-07-16

**Authors:** Hongbing Peng, Tiefeng Gong, Xiaoying Huang, Xun Sun, Hong Luo, Weizhong Wang, Junbiao Luo, Baowei Luo, Yanhui Chen, Xingxing Wang, Haifeng Long, Hua Mei, Chuang Li, Yanni Dai, Honghui Li

**Affiliations:** 1 Department of Respiratory Medicine, Loudi Central Hospital, No. 51, Changqing Middle Street, Loudi, 417000 People’s Republic of China; 2 Medical Rehabilitation Center, Loudi Central Hospital, No. 51, Changqing Middle Street, Loudi, 417000 People’s Republic of China; 3 Institute of Reproductive and Stem Cell Engineering, School of Basic Medical Sciences, Central South University, 172 Tongzipo Road, Changsha, 410013 People’s Republic of China; 4 National Engineering Research Center of Human Stem Cells, Lugu High-tech Zone, Changsha, 410000 People’s Republic of China; 5 Department of Respiratory and Critical Care Medicine, Second Xiangya Hospital, Central South University, No.139, Renmin Middle Road, Changsha, 410000 People’s Republic of China; 6 Department of Respiratory and Critical Care Medicine, The First Affiliated Hospital of Nanhua University, No. 69 Chuanshan Road, Hengyang City, 421001 People’s Republic of China; 7 Department of Critical Care Medicine, Second Affiliated Hospital of Nanhua University, No. 35 Jiefang Avenue, Xinxiang District, Hengyang City, 421001 People’s Republic of China

**Keywords:** Convalescent plasma, Mesenchymal stem cells, Coronavirus disease 2019, COVID-19, SARS-CoV-2

## Abstract

Acute respiratory distress syndrome virus-2 (SARS-CoV-2) responsible for coronavirus disease 2019 (COVID-19) infection, which causes global public health emergencies, has sped widely for more than 5 months and has the risk of long-term transmission. No effective treatment has been discovered to date. In the cases we report, the patient continued to deteriorate even after administration of antiviral drugs such as lopinavir/ritonavir, interferon-α, and ribavirin, as well as intravenous injection of meropenem, methylprednisolone, and immunoglobulin. So, we infused the patient with convalescent plasma (CP), and the absolute lymphocyte count increased the next day and returned to normal on the fourth day. Followed by intravenous infusion of mesenchymal stem cells (MSCs), bilateral infiltrates were absorbed and the pulmonary function was significantly improved. We note that the intravenous infusion of CP and MSCs for the treatment of severe COVID-19 patients may have synergistic characteristics in inhibiting cytokine storm, promoting the repair of lung injury, and recovering pulmonary function. We hope to provide a reference for the research direction of COVID-19 clinical strategies.

## Introduction

COVID-19 was first reported in Wuhan, China, in December 2019, with the characteristics of high infectivity and tall mortality. The virus caused a worldwide pandemic, and the World Health Organization declared a global public health emergency for novel coronavirus [1–3]. The cause of death of COVID-19 is virus-induced cytokine storm, with severe pulmonary injury, shock, acute respiratory disease syndrome (ARDS), and multiple organ dysfunction syndrome (MODS) [4]. Antiviral therapy and suppression of cytokine storms are two important directions of treatment. Specific treatments for COVID-19 are scarce. Therefore, identifying safe and effective therapies are essential for saving lives.

The convalescent plasma is a previous important means of treating infectious diseases and has received extensive attention. Convalescent plasma (CP) can effectively treat severe acute respiratory diseases caused by SARS-CoV, MERS-CoV, Ebola, H1N1, and other viruses [5, 6]. In the preliminary study of Zhang’s group [7], intravenous infusion of CP was given to patients with severe COVID-19, and 4 patients in the study recovered quickly, safe, and no adverse reactions. Neutralizing antibodies carried in convalescent plasma can reduce viral load, thereby reducing inflammation and improving survival [8].

MSCs have the ability of two-way immune regulation, which can inhibit excessive inflammation caused by microorganisms, thus inhibiting the immune damage of excessive inflammation to the pulmonary, liver, kidney, and heart [9, 10]. At present, in the treatment of COVID-19, some studies have shown that intravenous infusion of clinical-grade MSCs has achieved good efficacy, which benefits the strong immunoregulation function and endogenous repair ability of MSCs [11, 12]. The most important mechanism is that MSCs release many paracrine factors, such as micro-RNA, interacting with the immune response to exert immunoregulation and anti-inflammatory effects [13]. Adipose-derived mesenchymal stem cells (ASCs) with abundant exosomal microRNAs are used extensively in cellular therapies such as MSCs. Therefore, ASCs can also be used as an alternative treatment strategy for COVID-19 pneumonia [14]. The MSCs used in this case are freely derived from the National Engineering Research Center of Human Stem Cells, Changsha, Hunan, China, and belong to clinical-grade umbilical cord mesenchymal stem cells (UC-MSCs).

We reviewed a case of severe COVID-19 cured successfully with convalescent plasma-umbilical cord mesenchymal stem cells and observed and analyzed the change of clinical symptoms and laboratory data before and after treatment. We want to know whether there is a coordinated relationship between CP and MSCs in COVID-19 therapy. There are currently no relevant reports, to our knowledge. We hope to provide some references for the treatment of COVID-19.

## Methods

### Case presentation

A 66-year-old female patient suffered from cough, sore throat, and fever after contact with a confirmed case of COVID-19. On February 3 (illness day 10), oropharyngeal swab obtained from the patient tested positive for SARS-CoV-2 on quantitative real-time reverse transcriptase-polymerase chain reaction (RT-PCR) assays at the centers for disease control (CDC). She was admitted to the isolation ward for standard isolation treatment. On admission, the physical examination revealed a body temperature of 37.4 °C, 33 breaths per minute, blood pressure of 126/78 mmHg, and an oxygen saturation of 90% (indoor air). The patient’s oxygenation index was 243 mmHg (< 300 mmHg), the finger pulse oxygen saturation is less than 93%, and the respiratory rate was greater than 30 breaths per minute. According to the COVID-19 diagnosis and treatment guidelines, the patient is severe [15].

### Convalescent plasma and UC-MSCs

Convalescent plasma donors come from COVID-19 patients who meet the criteria for desegregation and discharge. They have the same blood type as the recipients, and written informed consent was obtained; donors who were checked for SARS-CoV-2, hepatitis B virus, hepatitis C virus, HIV, and syphilis are negative before collecting plasma. The convalescence plasma is collected by apheresis. Before infusion of convalescent plasma, the ELISA method was used to check that the anti-SARS-CoV-2-specific IgG antibody titer was greater than 1:160.

The UC-MSCs are provided freely by the National Engineering Research Center of Human Stem Cells. The MSCs are isolated and extracted from fetal umbilical cord without infectious diseases and pathological pregnancy. The cell products of MSCs were suspended in 100 mL of saline in strict accordance with standard operating procedures, and the total number of infused cells was 1 × 10^6^ cells per kilogram. The weight of the patient was 65 kg, the total number of cells was about 6.5 × 10^7^, and the ratio of live cells was 95.78%. All cell suspensions were prepared on the same day and stored at 4 °C. In order to maintain the maximum activity of cells, infusion was required within 12 h. The infusion is performed at a rate of about 40–55 drops/min for about 30–40 min, once every 3 days, and 3 times.

### Observed and measured variables

From admission to discharge, the researchers continue to observe and evaluate patients’ dynamic changes in clinical symptoms and laboratory results, especially after receiving plasma and stem cell therapy. The clinical, laboratory, and radiological results are recorded and confirmed by a team of trained doctors. We record possible adverse events, such as increased blood pressure and allergic reactions; the main therapeutic indicators observed are blood routine, C-reactive protein, IL-6, D-dimer, partial pressure of oxygen (PaO2), oxygenation index, chest CT, and clinical symptoms.

## Results

**Fig. 1:**
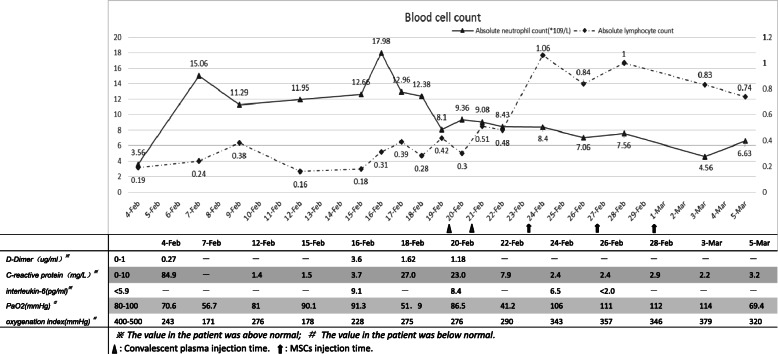
From the blood cell count curve, we noticed that there were two peaks of the absolute value of neutrophils (February 7 and February 16) and the low absolute value of lymphocytes before the peak (February 4 and February 15). In the fluctuation of these cell counts, CRP, D-dimer, PaO2, and oxygenation index all fluctuated, although the time did not coincide completely. At the second peak on February 16, IL-6 also increased significantly. It may be related to two cytokine storms. After the second cytokine storm, the absolute value of lymphocytes did not increase significantly after conventional treatment and increased after the application of convalescent plasma co-400 ml on February 20 and 21. After intravenous infusion of MSCs on February 24 and 27 and March 1, the patients could get rid of respiratory support and the symptoms of dyspnea were relieved

**Fig. 2:**
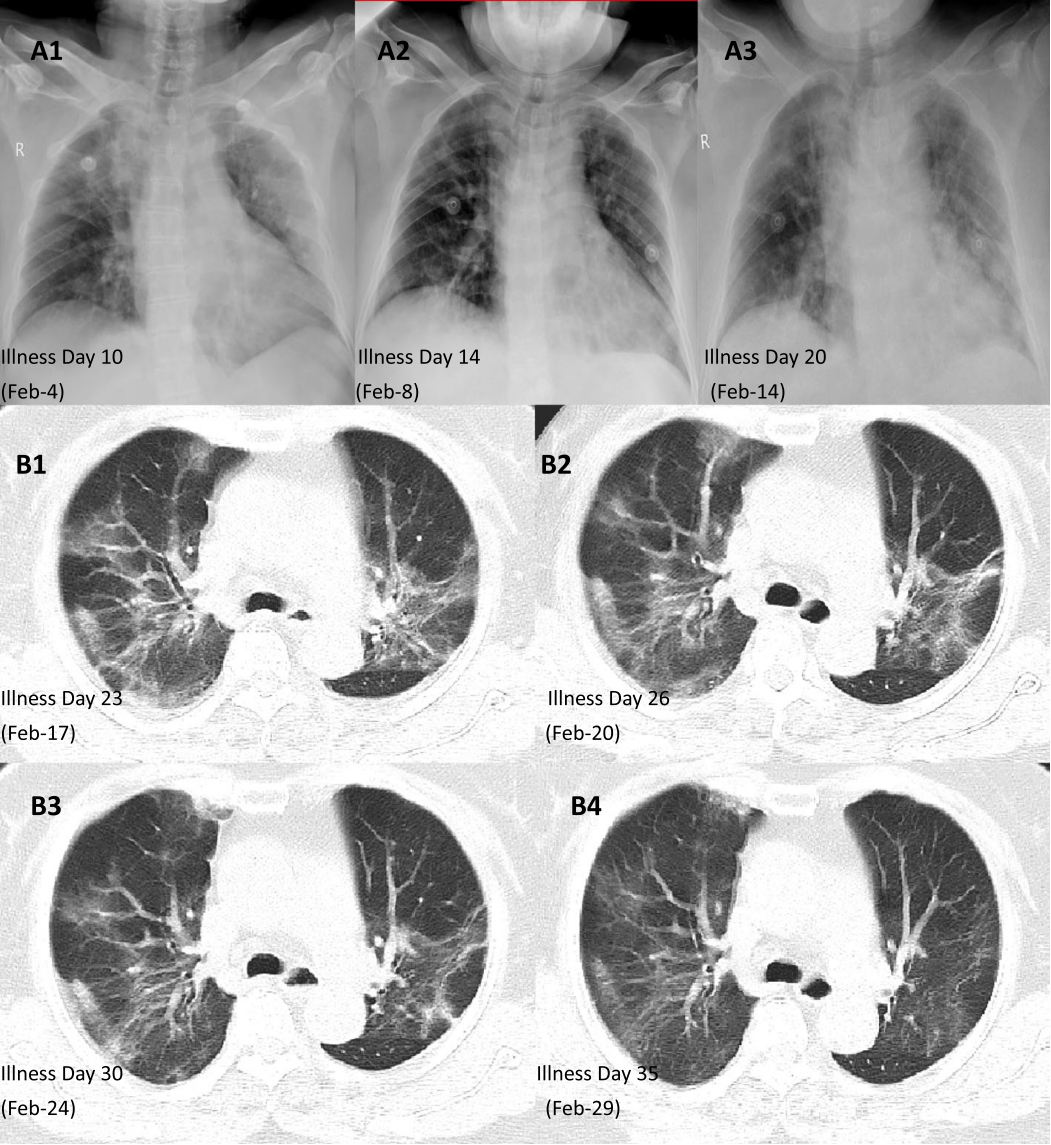
The chest X-ray (**A1**–**A3**) of the patient showed that the pulmonary exudative lesions improved after standard treatment and increased before the second cytokine storm. In the patient’s chest CT (**B1**–**B4**), we can see the absorption and evolution of pulmonary exudative lesions in patients with COVID-19; comparing February 20 and February 17, the pulmonary exudative lesions have no significant improvement. After the application of convalescent plasma, a small amount of exudative lesions was seen on the chest CT on February 24 (**B3**). On February 27, after the first application of umbilical cord mesenchymal stem cell therapy, pulmonary exudative lesions improved significantly (**B4**)

Routine laboratory testing is shown in Fig. 1. The absolute neutrophil count, C-reactive protein (CRP), D-dimer, IL-6, and related cytokine storm indicators showed twice peak changes, from February 4 to February 7 (illness day 10–14) and February 15 to February 16 (illness day 20–21); the oxygenation index had two troughs of 171 mmHg and 178 mmHg. At the same time, chest X-ray examination also showed repeated manifestations (see Fig. 2 A1, A2, and A3). The clinical results support twice cytokine storms that occurred during hospitalization.

After admission, following the COVID-19 diagnostic guidelines for treatment [15]. The symptoms and laboratory results have improved, but the absolute lymphocyte count did not return to normal (see Fig. 1) and has poor absorption of pulmonary exudative lesions (see Fig. 2 B1 and B2). Symptoms of dyspnea remain prominent. The patient still needs high-flow nasal cannula oxygen therapy (HFNC) combined with intermittent noninvasive ventilator-assisted ventilation. Therefore, convalescent plasma was injected twice on February 20 and 21 (illness days 27 and 28; total volume, 400 mL) as recommended in the convalescent plasma treatment guidelines [15]. On the fourth day after CP treatment, the absolute lymphocyte count returned to normal (see Fig. 1). However, the absorption of pulmonary exudative lesions is not obvious (see Fig. 2B2, B3), the symptoms of dyspnea have not been significantly improved, and HFNC are still required. So, there was a sympathetic application of UC-MSCs 3 times on February 24, February 27, and March 1. No infusion and allergic reactions, secondary infections, and adverse events were observed. On February 29 to review chest CT, the bilateral infiltration was obviously absorbed (see Fig. 2B4), and the nucleic acid test was negative on February 28 (illness day 35); the symptoms of dyspnea and dry cough improved significantly, and the endurance of daily activities improved. She recovered and discharged on March 6 illness day 42).

## Discussion

It has been more than 5 months since the first report of COVID-19, which has spread all over the world. At present, there is a comprehensive study of the clinical features of COVID-19 [4, 16, 17]. The study concluded that the clinical characteristics and routine laboratory data of patients with COVID-19 can also judge the occurrence and development of cytokine storm. Huang and his team [17] found that severe or critical illness often becomes worse 8 to 14 days after onset, with obvious dyspnea, a gradual decrease in lymphocyte count, and a significant increase in neutrophil count, CRP, and D-dimer, which is consistent with the characteristic changes of cytokine storms. On February 15–16, the neutrophil count, CRP, and D-dimer increased again, and chest imaging and symptoms in our case also supported twice cytokine storms. After routine treatment, the patient again experienced a cytokine storm; how to reverse it is a very thorny problem.

After the second cytokine storm, we adjusted the treatment schedule. The neutrophil count of the patients decreased gradually, but the absolute lymphocyte count did not return to normal (< 0.5 × 10^9^/L), which may be related to the persistent damage of SARS-CoV-2 to the immune system [17]. Application of specific neutralizing antibodies to remove viruses from patients will be a viable method. Convalescent plasma containing neutralizing antibodies was successfully treated for SARS [5]. Early application of CP therapy can inhibit a large number of virus replication, promote virus excretion, significantly shorten hospital stay, improve prognosis, and reduce mortality [6]. In severe SARS patients, the infusion of CP reduces the viral load to zero the next day [18]. In our case, the lymphocytes began to rise the day after CP infusion and returned to normal on the third day, which may be related to the elimination of the virus. Meanwhile, the symptoms of dyspnea improved, the activity capacity increased more than before, and the oxygenation index was > 300 mmHg the next day. A recent study used CP to treat 5 patients with COVID-19, all of which achieved good results; among them, 3 patients did not need mechanical ventilation within 2 weeks [19]. For current limited means of inhibiting SARS-CoV-2, CP is a good choice for the treatment of COVID-19, if the disease cannot be controlled and continues to progress [20]. Presently, the conclusion of the efficacy of CP for COVID-19 comes from a small sample study [19, 21], and studies with larger samples are required. The ultimate cause of death in critically ill COVID-19 patients is associated with multiple organ failure due to cytokine storms, and CP cannot completely reverse this process [22]. So, regarding the mechanism of CP in the treatment of COVID-19, the unanimous conclusion is to promote the clearance of the virus, and whether to reduce the mortality rate is still controversial.

With convalescent plasma treatment, the symptoms of dyspnea improved, and the non-invasive ventilator weaned, but the patient still needed HFNC for treatment. After pulmonary rehabilitation exercise, the pulse oxygen saturation was as low as 85%, and there are still more exudative lesions in the bilateral lung; it may be associated with the persistent immune damage caused by cytokine storm, resulting in pulmonary diffusion dysfunction [23]. After completing the intravenous infusion of clinical-grade UC-MSCs twice, the chest CT was rechecked on February 29 (illness day 36), and bilateral infiltrates were clearly absorbed. In terms of clinical symptoms, we noticed that the daily activities were not restricted, as well as the patient successfully discontinued HFNC and only inhaled through the nasal catheter. This may be related to MSCs improving the pulmonary microenvironment, promoting the endogenous repair of the host, and repairing pulmonary damage caused by inflammation [24, 25], thereby restoring lung diffusion function and improving pulmonary function. Gentile and Sterodimas [13, 14] analysis shows that ASCs and stromal vascular fraction cells (SVFs) can improve the microenvironment because they can secrete pro-angiogenic factors, such as vascular endothelial growth factor (VEGF) and platelet-derived growth factor (PDGF), to establish a new microvascular network in the damaged tissue to provide nutrition and oxygen, and promote tissue repair. MSCs play a role in antiviral pneumonia by including paracrine factors, exocrine vesicles, and mitochondrial transfer, which can inhibit the inflammatory response and avoid cytokine storms; it can also reduce and clear alveolar effusion to reduce pulmonary edema [26]. Before and after treatment with UC-MSCs, our researchers continuously monitored the patient’s blood cell count, IL-6, oxygenation index, and PaO2 and noticed a steady decline in absolute neutrophils count and IL-6; absolute lymphocyte count, the oxygenation index, and PaO2 gradually increase. Laboratory data indicate that UC-MSCs regulate the immune response, inhibit the occurrence of cytokine storms, and repair lung tissue. Recently, Dr. Zhao’s team completed a small sample study on MSCs in the treatment of patients with coronavirus pneumonia, and the results of the study support the safe and effective treatment of coronavirus pneumonia by intravenous MSCs transplantation, especially for critically ill patients [11]. A team headed by Dr. Atluri [9] considered that UC-MSCs can suppress cytokine storm, a key factor that causes acute exacerbation and death of COVID-19, and supports the use of UC-MSCs. Prospective multicenter studies of UC-MSC therapy for COVID-19 are ongoing [27] and are expected to yield promising results.

## Conclusions

Although it has been observed from one case that intravenous infusion of plasma and mesenchymal stem cells have a synergistic therapeutic effect on patients with severe COVID-19 pneumonia, in theory, the mechanisms of action of convalescent plasma and mesenchymal stem cells have complementary characteristics. We believe that this treatment strategy might benefit patients with COVID-19 pneumonia without better options.

## Data Availability

The data that support the findings of this study are available from the corresponding author upon reasonable request.

## Abbreviations

SARS-CoV-2: Acute respiratory distress syndrome virus-2
COVID-19: Coronavirus disease 2019
ARDS: Acute respiratory disease syndrome
MODS: Multiple organ dysfunction syndrome
SARS-CoV: Severe acute respiratory syndrome coronavirus
MERS-CoV: Middle East respiratory syndrome coronavirus
H1N1: Novel 2009 influenza A
CP: Convalescent plasma
MSCs: Mesenchymal stem cells
UC-MSCs: Umbilical cord mesenchymal stem cells
RT-PCR: Real-time reverse transcriptase polymerase chain reaction
CDC: The centers for disease control
ICU: Intensive care unit
HFNC: High-flow nasal cannula oxygen therapy
CRP: C-reactive protein
IL-6: Interleukin-6
PaO2: Pressure of oxygen
ASCs: Adipose-derived mesenchymal stem cells
SVFs: Stromal vascular fraction cells
VEGF: Vascular endothelial growth factor
PDGF: Platelet-derived growth factor
